# Injection Botulinum Toxin A in Treatment of Resistant Chronic Low Back Pain: A Prospective Open-Label Study

**DOI:** 10.7759/cureus.17811

**Published:** 2021-09-08

**Authors:** Jagannatha Sahoo, Debasish Jena, Amrutha Viswanath, Apurba Barman

**Affiliations:** 1 Physical Medicine and Rehabilitation, All India Institute of Medical Sciences, Bhubaneswar, IND

**Keywords:** low back pain, pain, botulinum toxin a, botox, neurotoxin

## Abstract

Objective

The aim of this study was to evaluate the effect of botulinum toxin A (BTX-A) injection on patients with chronic low back pain (CLBP).

Design

In this open-label prospective study, patients with CLBP who satisfied inclusion and exclusion criteria received 100 units of BTX-A injection. Patients were followed up at four weeks, three months, and six months after injection. Pain and function were assessed with visual analog scale (VAS), Roland-Morris Disability Scale (RMS), and Oswestry Disability Index (ODI) at baseline and subsequent visits.

Results

A total of 19 participants with a mean age of 41.11 years completed the study. Compared to baseline, a significant improvement in all scores was observed that persisted up to six months post-injection (P<0.001). Only two patients reported transient injection site pain that improved over two to three days without any treatment.

Conclusion

BTX-A injection is safe and improves pain and function in patients with resistant CLBP. The effects are more beneficial when the population is more homogenous in diagnosis and devoid of negative predictors for the outcome.

## Introduction

Low back pain (LBP) is a major public health problem worldwide and is a significant cause of morbidity and reduced quality of life [1,2]. Among individuals with acute LBP, 5% to 10% may develop chronic low back pain (CLBP) which impairs psychosocial, vocational, avocational, and behavioral measures of disability [3]. A multitude of pathologies is responsible for CLBP including chronic muscle spasm, degenerative spine changes, disc herniation, spondylolisthesis, and facet joint inflammation.

Despite several available treatment options, relief is often inadequate and adverse effects of analgesics and opioid medications limit their chronic use. The lumbar erector spinae muscles have become an important therapeutic target as an increased contraction of these muscles has been linked to lumbar stiffness hence the intensity of LBP [4]. Many therapeutic options targeting these local muscles are being used including muscle relaxants, massage, exercise, physical modalities, and botulinum toxin (BTX) injection. Even though surgery can effectively treat root pain rather than alleviate LBP itself, reports of failed back surgery syndrome (FBSS) are not uncommon [5]. Nevertheless, studies have demonstrated that effective intervention can reduce healthcare costs and morbidity in persons with CLBP [6].

BTX-A is one of the most potent neurotoxins which is produced by Clostridium botulinum. It is a potent inhibitor of acetylcholine (Ach) release from presynaptic vesicles. BTX-A effectively treats spasticity, dystonia, and several other forms of muscle spasm, including blepharospasm [7]. Literature depicting the role of BTX-A on CLBP are still scarce and inconsistent in their methodology and results. Therefore, the effect of BTX-A on CLBP remains a matter of debate and needs further good quality trials.

## Materials and methods

This prospective open-label study was conducted in the Department of Physical Medicine and Rehabilitation, in a tertiary care institute of India. The ethical approval has been taken from Institutional Research Cell, AIIMS, Bhubaneswar (Registration no- ECR/534/Inst/OD/2014/RR-17) on 23rd January 2018. The IRB approval number was AIIMS/BBSR/RC/130/2017, dated 23rd January 2018. After ethical approval, we started recruiting patients (total-19) for our study. Inclusion criteria were: (1) chronic and stable LBP for at least six months; (2) age 18-70 years; (3) failure of medical and/or surgical treatments. Exclusion criteria were: (1) chronic LBP attributable to acute and/or serious pathologies including fracture, infection, and neoplasm; (2) systemic inflammation; (3) pregnancy (current or planned) and/or breastfeeding; (4) history of neuromuscular junction disorders; (5) primary muscle weakness; (6) known allergy or sensitivity to BTX-A; (7) history of any psychiatric illness; (8) any history of injection to paravertebral muscles in last six months and (9) patients who were not motivated to undergo the treatment. Patients were advised against any change in the current treatment regimen for LBP. All patients who satisfied the inclusion and exclusion criteria were explained about the study and included in the trial after signing the informed consent form.

All patients were evaluated at baseline, at four weeks, at three months, and at six months post-injection. During each evaluation, patients were assessed with visual analog scale (VAS) for pain, Roland-Morris Disability Scale (RMS), and Oswestry Disability Index (ODI) for disability. Participants rated their average perception of LBP during the past one month on VAS having a 10-cm linear axis with a left end-point of “no pain” and right end-point of “worst pain ever”. The pain was recorded by measuring the distance on the 10-point scale [8]. Participants also completed the RMS questionnaire by placing a checkmark beside the statement if it applies to them that day. The RMS is a 24-item questionnaire that was calculated by adding up the number of checked items and the scores range from 0 (no disability) to 24 (maximum disability) [9]. Lastly, participants were asked to complete the ODI, a 10-item scale covering two subsections, i.e., pain and daily function. Each item is rated on a six-point scale (0-5), and the total score ranged from 0 (no disability) to 100 (highest disability) [10].

BTX-A (Botox, Allergan Inc.), with a concentration of 50 units/mL, was prepared by reconstitution of the frozen-dried toxin (100 units) with normal saline (2 ml) and was drawn in a 1-cc tuberculin syringe. The injection was performed with a 25 gauze needle and the needle size was selected depending upon the body habitus of the subject. The intervening physician injected the solution of 12.5 units each at 4 sites per side (L1 to S1) either by locating points of maximum tenderness or at equidistant sites to encompass the whole length of the lumbar paraspinal muscle. The injection was performed without any guidance as to the superficial location and the adequate size of the target muscle made it easily accessible. The total injected dose of BTX-A in a single participant didn’t exceed 100 units. Patients were advised to report any adverse events during the study period and to follow-up at four weeks, three months, and six months post-injection as per study protocol.

The statistical analysis was done with the help of SPSS software version 20.0 (Armonk, NY: IBM Corp.). The categorical variables were presented as n (%) and continuous data as mean and standard deviation (SD). The significance of outcome measures (VAS, RMS & ODI) at follow-up visits compared to baseline was determined by the paired t-test.

## Results

A total of 21 patients were included in the study but only 19 patients completed the study with a mean age of 41.11 years. Among them, 10 were male and nine were female patients. The mean duration of CLBP was 38.58 months. Radiation of LBP into lower limbs was present in five patients (26.32%) with three of them having both limb pain and two patients having only single limb pain. Rest 14 patients (73.7%) had isolated LBP without any radiation. LBP was unilateral in four patients and bilateral in 15 patients. Fifteen patients took one or more non-opioid pain medications, and four patients used heat modalities for pain relief. None of them were using any opioid pain medications. MRI of the lumbosacral spine was abnormal in 12 patients with common findings being degenerative changes, disc herniation, lumbar spinal stenosis, spondylolisthesis, or a combination of these abnormalities. Five patients were diagnosed with myofascial pain syndrome (MPS) with palpable trigger points. No patients were having any neurologic deficits, any history of back surgery, any red flag or yellow flag signs. Table 1 summarizes demographic data and baseline characteristics of all participants.

**Table 1:** Demographic and baseline clinical data of participants. LBP: low back pain; MRI: magnetic resonance imaging; MFPS: myofascial pain syndrome.

Number of participants	19
Mean age in years (range)	41.11 (27-62)
Male/female	10/9
Mean duration of LBP in months (range)	38.58 (6-180)
Radicular Pain (%)	5 (26.32%)
Unilateral Pain (%)	4 (21.05%)
Bilateral Pain (%)	15 (78.95%)
Patients with abnormal MRI findings (%)	12 (63.16%)
Patients with lumbar spondylosis (%)	5 (26.32%)
Patients with spondylolisthesis (%)	3 (15.79%)
Patients with disc herniation (%)	9 (47.37%)
Patients with MFPS (%)	5 (26.32%)
Patients on pain medications (%)	15 (78.95%)

**Figure 1:**
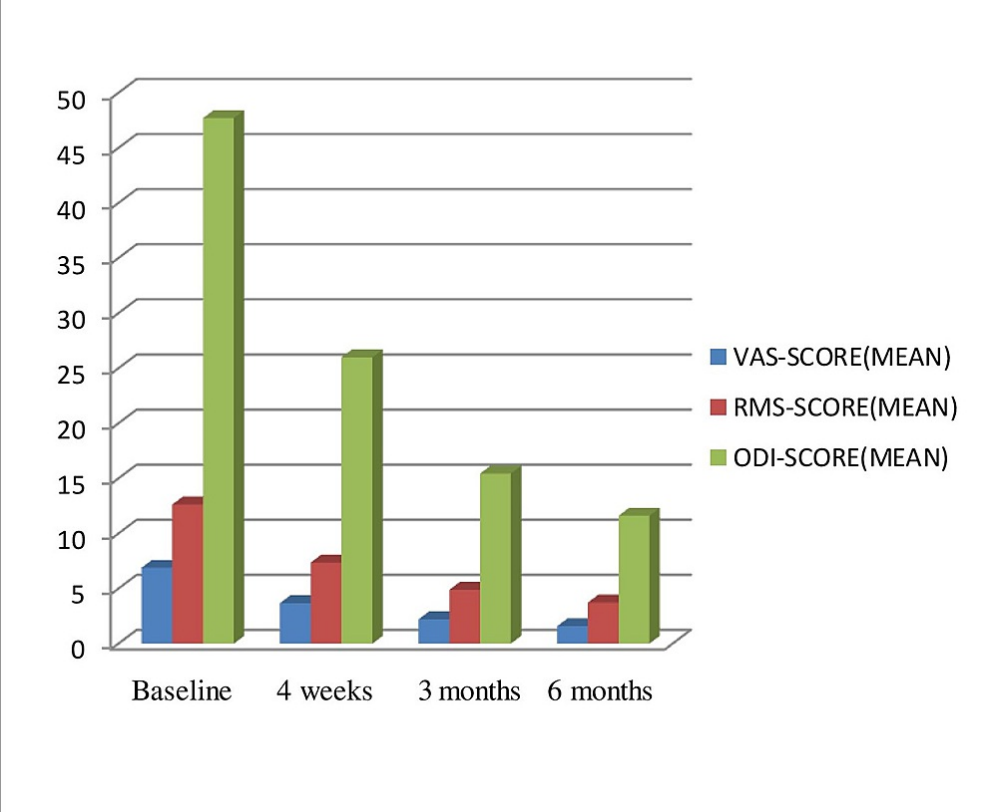
Comparison of mean baseline and subsequent post-injection scores. VAS: visual analog scale; RMS: Roland-Morris Score; ODI: Oswestry Disability Index. P-value is significant for all comparisons to baseline (P<0.001).

At baseline, the mean average VAS score was 6.89, the mean RMS score was 12.63, and the mean ODI score was 47.70. Among all participants, 18 patients significantly reduced all scores at subsequent follow-ups that persisted up to 6 months post-injection. The percentage reduction in scores from baseline was also significant across all subsequent visits. Only one patient didn’t respond to injection which might be attributed to the presence of multiple etiology. In all responders, the beneficial response to BTX-A occurred within 24-72 hours of injection. No patient reported worsening in pain and/or function after administration of BTX-A. Only two patients reported side effects as local injection site pain which subsided without treatment in two to three days. Comparison of outcome scores with baseline at all follow-ups has been summarized in Figure 1 and Table 2.

**Table 2:** Mean baseline and subsequent post-injection scores. VAS: visual analog scale; RMS: Roland-Morris Score; ODI: Oswestry Disability Index. All the values within parentheses indicate the percentage reduction in the score from baseline; P-value is significant for all comparisons to baseline (P<0.001).

	Baseline	Four weeks	Three months	Six months
VAS (mean±SD)	6.89±1.41	3.68±1.73 (46.59%)	2.21±1.84 (67.92%)	1.58±1.64 (77.21%)
RMS (mean±SD)	12.63±2.41	7.37±3.89 (41.65%)	4.89±3.29 (58.12%)	3.74±2.90 (70.39%)
ODI (mean±SD)	47.70±15.65	25.99±17.65 (45.51%)	15.44±10.72 (67.63%)	11.61±10.54 (75.66%)

## Discussion

Paraspinal muscle hyperactivity has been implicated as an important contributor to CLBP. The source of the pain can be the ischemic muscle itself due to the accumulation of metabolic waste products or from the adjacent tissues such as ligaments, tendons, and joints which are under continuous stress by the hyperactive muscles. Also compared to subjects without pain, patients with CLBP demonstrated increased electrical activity of the paraspinal muscles in electromyographic studies [11]. This provides the rationale for therapeutic options aiming at reducing the tone of the hyperactive muscles.

BTX-A has demonstrated a mild to moderate analgesic effect in various chronic musculoskeletal pain conditions besides its well-known tone reducing effect. In a recent meta-analysis, it has been found to be particularly efficacious in plantar fasciitis, tennis elbow, and back pain [12-14]. MPS is considered an important contributor in developing CLBP. In some past trials, BTX-A injection directly into trigger points in subjects with MPS has produced 72% to 80% improvement in symptoms [15-17].

According to the American Pain Society Clinical Practice Guidelines [18], there was insufficient evidence to evaluate the merits of BTX injections for LBP properly. The European guidelines for the management of chronic nonspeciﬁc LBP [19], concluded that there is limited evidence for BTX injections in the treatment of chronic non-specific LBP. The International Centre for Allied Health Evidence (2017) conducted a systematic review of BTX injection for LBP. On the basis of results, they recommended the use of BTX-A in CLBP for a short-term (two to three months post-injection) effect both on pain relief and functional outcome [20].

Ney et al [21], conducted an open-label prospective trial on 60 patients with CLBP and reported a significant beneficial effect of BTX-A at three weeks in 60% and at two months in 58% of the patients with LBP and radicular pain. A sustained beneﬁcial eﬀect from the ﬁrst injection was also noted in a significant minority of subjects at four (16.6%) and six months (8.3%). Similar trials in the past have produced inconsistent results. They are limited by one or more of the following factors: short duration of follow-up, heterogeneity of diagnosis among participants, lack of control group, small sample size, and lack of cost-benefit analysis. This draws attention for further research focusing on the above factors and to provide a more precise conclusion.

In contrast, our study's improvement in pain and function persisted up to 6 months post-injection and can be attributed to a couple of the following important factors. Firstly, participants were devoid of certain conditions that might have negatively affected the outcome including failed back surgery syndrome, yellow and red flag signs, any acute or serious pathology, and lack of motivation to undergo the intervention. Similar studies in the past have not considered excluding the above factors which might have contributed to the short-term effects of BTX-A. Secondly, all participants were aware of the high cost of BTX-A and the interventional nature of the treatment before giving their consent for participation. This may have led to a positive pre-conception about the treatment outcome and possibly could have contributed to a placebo response alone, or in combination with the actual therapeutic response.

The analgesic effect of BTX-A can be attributed to its effect on various nociception-inducing mechanisms. It inhibits the release of a number of neurotransmitters (NTs) from presynaptic vesicles which include acetylcholine, substance P, bradykinin, calcitonin gene-related peptide, and glutamate [22]. This inhibition of NTs mediating nociception from peripheral nerve endings and dorsal root ganglion along with a reduction in local inflammation is known to be the major mechanism responsible for the analgesic properties of BTX. In addition, BTX also reduces allodynia and hyperalgesia by reducing sensitization of the wide dynamic range (WDR) neurons in the spinal cord which occurs by reduced intrafusal muscle spindle discharges [23]. Other mechanisms responsible for the analgesic effect may also include a reduction of the sympathetic transmission, a direct analgesic effect of BTX-A metabolites, and an indirect effect on spinal cord neurons [24].

Similar to our findings, a favorable safety and tolerability profile has been shown across a wide spectrum of therapeutic uses with focal weakness being the only reported adverse event [25]. Two participants in our study developed local injection site pain which subsided within two to three days of injection without any treatment. This is in accordance with the reports of similar past trials.

This study despite having encouraging results is not without limitations. First, the sample size was small which prevents the generalizability of the results. Second, the lack of a control group and non-blinding may reduce the study's internal validity and causal inference cannot be established. Nevertheless, the number of patients receiving BTX-A is similar to that in past trials, which also reported a significant positive effect on CLBP. Our study adds to the findings of previous trials by showing a superior effect of BTX-A on CLBP when the population subset is more homogenous in their diagnoses. Therefore we encourage future trials on a larger cohort with a superior study design to further support our results.

## Conclusions

This study demonstrated a more beneficial and persistent effect of BTX-A injection on CLBP when the patient group is more homogenous in their diagnoses. Also, BTX-A appears like a safer alternative to manage chronic and refractory LBP with only very few and transient side effects. However, further good quality trials with a larger sample size are needed to support this conclusion. Also, future trials should focus on the cost-benefit analysis of BTX to warrant its wider clinical application.
